# The complete chloroplast genome of *Bupleurum marginatum* var. *stenophyllum* (H. Wolff) Shan & Yin Li (Apiaceae), a new substitution for Chinese medicinal material, Bupleuri Radix (Chai hu)

**DOI:** 10.1080/23802359.2020.1870899

**Published:** 2021-02-09

**Authors:** Gaixia Zhang, Hui Wang, Jiemei Jiang, Qiuling Wang, Baoli Li, Linchun Shi, Jianhe Wei

**Affiliations:** a Institute of Medicinal Plant Development, Chinese Academy of Medical Sciences and Peking Union Medical College, Beijing, China; b Institute of Sericulture, Chengde Medical University, Chengde, China

**Keywords:** *Bupleurum marginatum* var. *stenophyllum*, complete genome, phylogenetic analysis, substitution, discrimination

## Abstract

The root of *Bupleurum marginatum* var. *stenophyllum* (H. Wolff) Shan & Yin Li (Apiaceae), a new substitution for the popular Chinese medicinal material, Bupleuri Radix (Chai hu), is not easily distinguishable via traditional methods. The complete chloroplast genome sequence of *B. marginatum* var. *stenophyllum* was characterized using next-generation sequencing and the *de novo* assembly method. The complete genome was 155,576 bp in length and contained two inverted repeat (IR) regions of 26,311 bp, a large single-copy (LSC) region of 85,351 bp, and a small single-copy (SSC) region of 17,603 bp. It encoded 113 unique genes consisting of 79 protein-coding genes (PCGs), 30 transfer RNA genes, and four ribosomal RNA genes. Importantly, three genes (*petB*, *petD* and *rps16*) with small exon, and one trans-splicing gene (*rps12*) were correctly annotated. The overall GC content of the *B. marginatum* var. *stenophyllum* chloroplast genome is 37.7%. The phylogenetic analyses indicated that *B. marginatum* var. *stenophyllum* was closely related to *B. marginatum*. Moreover, many genetic information sites were available for distinguishing *B. marginatum* var. *stenophyllum* from the official ‘Chai hu’ plant sources, *B. scorzonerifolium* Willd. and *B. chinense* DC.

*Bupleurum marginatum* var. *stenophyllum* (H. Wolff) Shan & Yin Li is a perennial plant belonging to *Bupleurum* L. of the family Apiaceae (Wu et al. 2005). The roots of this species are often referred to as ‘Zang chai hu’ in the literature and frequently used in folk medicine for the treatment of various diseases, such as cough, fever, and influenza in Tibet, as well as the Yunnan, Guizhou, and Gansu provinces of China (Fang et al. 2017; Guo et al. 2018). Considering the popularity of Bupleuri Radix (Chai hu) in the current medicinal market, as well as the stable supply and substantial yield, ‘Zang chai hu’ represents a commercial source for regional substitutes of ‘Chai hu’ or other local medicinal purposes (Guo et al. 2018). Even though the resources, phytochemistry, pharmacology, and related research associated with this medicine are increasing (Ding et al. 2016; Fang et al. 2017; Yuan et al. 2017), no comprehensive genomic analysis currently exists. Therefore, the chloroplast genome of *B. marginatum* var. *stenophyllum* and the analysis of its genetic information and phylogenetic relationships within the Apiaceae family are explored here to improve the development and utilization of this resource.

Fresh *B. marginatum* var. *stenophyllum* leaf materials were collected from Lintao County, Gansu Province, China (N35°14′5.67″, E103°44′1.44″), and allocated the ID, GSC03. A voucher specimen was deposited in the herbarium of the Institute of Medicinal Plant Development, Chinese Academy of Medical Sciences and Peking Union Medical College, with an assigned voucher number of HPCH0002. The code of this herbarium was denoted by ‘IMD’ (NYBG: https://www.nybg.org/). A CTAB-based extraction method, modified according to a technique delineated by Porebski et al. (Porebski et al. 1997), was employed to extract total genomic DNA from the fresh leaf samples. A NanoDrop 2000 ultra-micro spectrophotometer was used to measure the quality of the DNA (Thermo Fisher Scientific Inc., USA), and next quantified using Qubit 4.0 (Thermo Fisher Scientific Inc., USA). This process was followed by creating ∼350 bp PCR-free libraries from the extracted genomic DNA according to the TruSeq DNA PCR-free library preparation guide. Sequencing was performed using the Illumina NovaSeq platform, generating 5,669,517 paired-end reads, totaling 1.7 Gb. Trimmomatic v0.38 (Bolger et al. 2014) was employed to filter the low-quality reads and the sequencing adapter. A NOVOPlasty toolkit (Dierckxsens et al. 2016) was used to compile the complete *B. marginatum* var. *stenophyllum* chloroplast genome after which the CPGAVAS2 webserver was employed for separate annotation (Shi et al. 2019).

The complete chloroplast genome of *B. marginatum* var. *stenophyllum* displayed a total sequence length of 155,576 bp (GenBank accession no. MT075712) and a GC content of 37.7%, which was similar to that of other *Bupleurum* genus species, such as *B. chinense* DC (Zhang et al. 2019). Furthermore, it featured the typical quadripartite structure of most angiosperm chloroplasts, containing two IR regions of 26,311 bp, an LSC region of 85,351 bp, and an SSC region of 17,603 bp. Finally, a total of 113 unique genes were successfully annotated in the complete chloroplast genome of *B. marginatum* var. *stenophyllum*, comprising 79 PCGs, 30 transfer RNA genes, and four ribosomal RNA genes. 17 unique genes (11 PCGs and six transfer RNA genes) contain introns. Three genes (*petB*, *petD,* and *rps16*) have small exon, and one gene (*rps12*) has been recognized as a trans-splicing gene. Codon usage analysis showed that 64 types of codons were used for the 79 PCGs and the AAA codon encoding isoleucine was the most frequently used.

The phylogenetic position of *B. marginatum* var. *stenophyllum* was confirmed using RAxML *v*8.0.0 (Stamatakis 2014) to create a maximum-likelihood tree (with 1000 bootstrap replicates) using the GTR + I + G model based on its and other 37 chloroplast genomes (Figure 1). The *B. marginatum* var. *stenophyllum* was closely clustered with *B. marginatum* and significantly separated from other species, including the official plant sources of ‘Chai hu,’ *B. scorzonerifolium* Willd. and *B. chinense* DC. The phylogenetic analysis results were consistent with the morphological observations, indicating that *B. marginatum* var. *stenophyllum* represented a separate species (Shan and Li 1974). Furthermore, the recent comparative analysis of chemical constituents in *B. chinense* and *B. marginatum* var. *stenophyllum* demonstrated that the compounds in *B. marginatum* var. *stenophyllum* differed from those in *B. chinense* (Yang et al. 2019). Therefore, substituting *B. marginatum* var. *stenophyllum* is a potential risk for using Chinese patent medicines containing ‘Chai hu’ (Liu et al. 2019). In conclusion, the complete chloroplast genome of *B. marginatum* var. *stenophyllum* provides essential DNA data for distinguishing *B. marginatum* var. *stenophyllum* from *B. scorzonerifolium*, and *B. chinense*.

**Figure 1.**
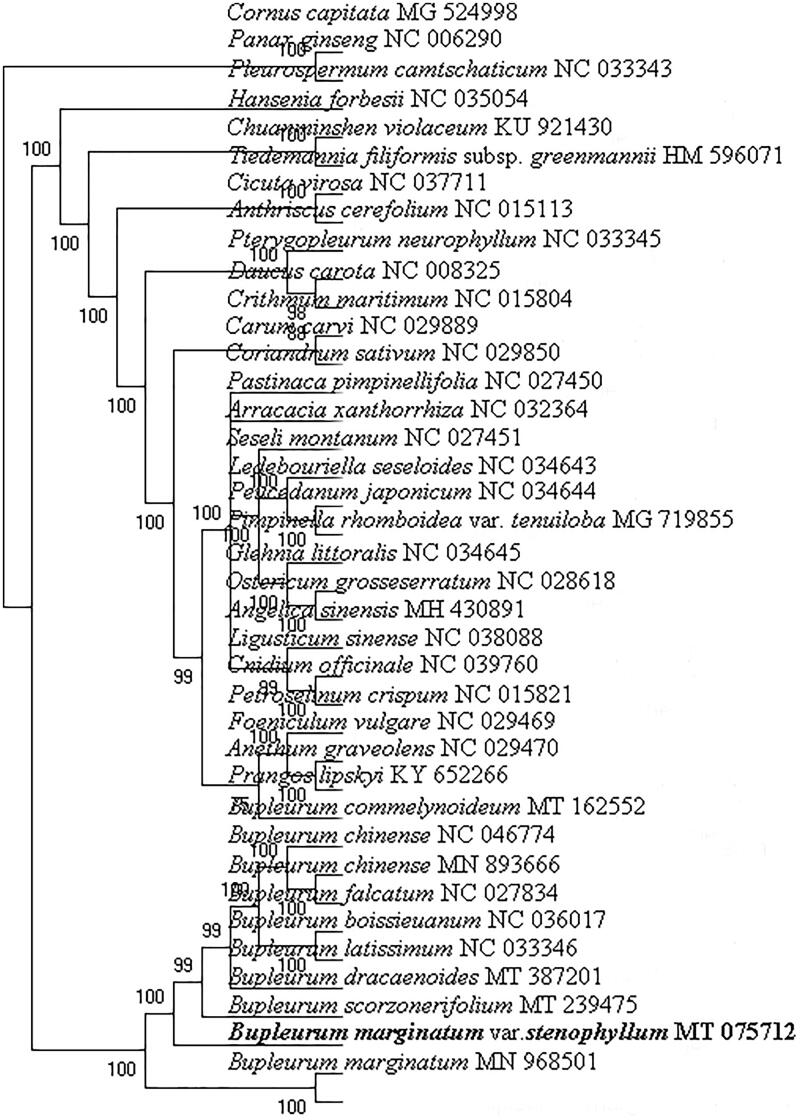
The phylogenetic position for *B. marginatum* var. *stenophyllum* according to the ML phylogenetic tree constructed based on 38 chloroplast genomes. The bootstrap support values are shown on the branches.

## Data Availability

The genome sequence data that support the findings of this study are openly available in GenBank of NCBI at (https://www.ncbi.nlm.nih.gov/) under the accession no. MT075712. The associated BioProject, SRA, and Bio-Sample numbers are PRJNA682316, SRR13195839, and SAMN16990902, respectively.
